# Balancing accuracy and efficiency: co-design of hybrid quantization and unified computing architecture for spiking neural networks

**DOI:** 10.3389/fnins.2025.1665778

**Published:** 2025-10-15

**Authors:** Jiahao Li, Ming Xu, Heng Dong, Bin Lan, Yuxin Liu, He Chen, Yin Zhuang, Yizhuang Xie, Liang Chen

**Affiliations:** ^1^National Key Laboratory of Space-Born Intelligent Information Processing, Beijing Institute of Technology, Beijing, China; ^2^Sichuan Tianfu New Area, Beijing Institute of Technology Innovation Equipment Research Institute, Chengdu, China; ^3^Beijing Institute of Technology Chongqing Innovation Center, Chongqing, China

**Keywords:** spiking neural networks, quantization, field-programmable gate array, algorithm-hardware co-design, unified processing elements, resource-constrained devices

## Abstract

The deployment of Spiking Neural Networks (SNNs) on resource-constrained edge devices is hindered by a critical algorithm-hardware mismatch: a fundamental trade-off between the accuracy degradation caused by aggressive quantization and the resource redundancy stemming from traditional decoupled hardware designs. To bridge this gap, we present a novel algorithm-hardware co-design framework centered on a Ternary-8-bit Hybrid Weight Quantization (T8HWQ) scheme. Our approach recasts SNN computation into a unified “8-bit × 2-bit” paradigm by quantizing first-layer weights to 2 bits and subsequent layers to 8 bits. This standardization directly enables the design of a unified PE architecture, eliminating the resource redundancy inherent in decoupled designs. To mitigate the accuracy degradation caused by aggressive first-layer quantization, we first propose a channel-wise dual compensation strategy. This method synergizes channel-wise quantization optimization with adaptive threshold neurons, leveraging reparameterization techniques to restore model accuracy without incurring additional inference overhead. Building upon T8HWQ, we propose a novel unified computing architecture that overcomes the inefficiencies of traditional decoupled designs by efficiently multiplexing processing arrays. Experimental results support our approach: On CIFAR-100, our method achieves near-lossless accuracy (<0.7% degradation vs. full precision) with a single time step, matching state-of-the-art low-bit SNNs. At the hardware level, implementation results on the Xilinx Virtex 7 platform demonstrate that our unified computing unit conserves 20.2% of lookup table (LUT) resources compared to traditional decoupled architectures. This work delivers a 6 × throughput improvement over state-of-the-art SNN accelerators—with comparable resource utilization and lower power consumption. Our integrated solution thus advances the practical implementation of high-performance, low-latency SNNs on resource-constrained edge devices.

## 1 Introduction

A fundamental tension exists between the escalating computational demands of sophisticated artificial neural networks (ANNs) and the stringent computing, storage, and power constraints inherent to edge devices (Hinton et al., 2015; Li et al., 2017; Wei et al., 2024). Although ANNs demonstrate exceptional performance across diverse computational tasks, their reliance on extensive model sizes and dense multiply-accumulate (MAC) operations inherently leads to prohibitively high energy consumption and significant computational latency. As a promising solution, brain-inspired Spiking Neural Networks (SNNs) encode and transmit information through sparse spiking signals (Maass, 1997), enabling hardware-level computational sparsity (Plagwitz et al., 2023). When implemented on reconfigurable platforms such as field-programmable gate arrays (FPGAs), this sparsity inherently bypasses redundant operations, drastically reducing dynamic power consumption and enabling high-performance edge AI systems (Karamimanesh et al., 2025).

The practical deployment of SNNs is fundamentally at odds with critical performance and hardware limitations. A primary impediment is the persistent accuracy gap. SNN training algorithms, while advancing, have yet to consistently match the performance of structurally equivalent ANNs (Luo et al., 2025; Su et al., 2023). Furthermore, unlocking the profound energy efficiency of SNNs is contingent upon a transition from inefficient von Neumann-based simulations to specialized hardware, such as FPGAs and neuromorphic chips (Karamimanesh et al., 2025). In this context, model quantization represents a pivotal strategy (Jacob et al., 2018; Courbariaux et al., 2015). By reducing the bit width of model weights, quantization can drastically curtails storage requirements and computational complexity, a crucial step for adapting SNNs to these resource-constrained hardware platforms.

Current research in SNN quantization is bifurcated into two distinct trajectories, yet both converge on a significant, unresolved hardware implementation challenge. The first path involves moderate quantization to 8-bit or 4-bit precision, a strategy that achieves effective model compression while maintaining performance, but offers limited optimization for ultra-resource-constrained environments (Zou et al., 2024; Putra and Shafique, 2021; Chowdhury et al., 2021). The second, more aggressive approach utilizes low-bit quantization, such as binary (1-bit) (Cao et al., 2025; Eshraghian and Lu, 2022) or ternary (~2-bit) schemes (Hasssan et al., 2024). This method dramatically reduces hardware complexity by converting multiplications into efficient bitwise operations or additions, though it frequently incurs severe accuracy degradation (Zou et al., 2024).

**Figure 1:**
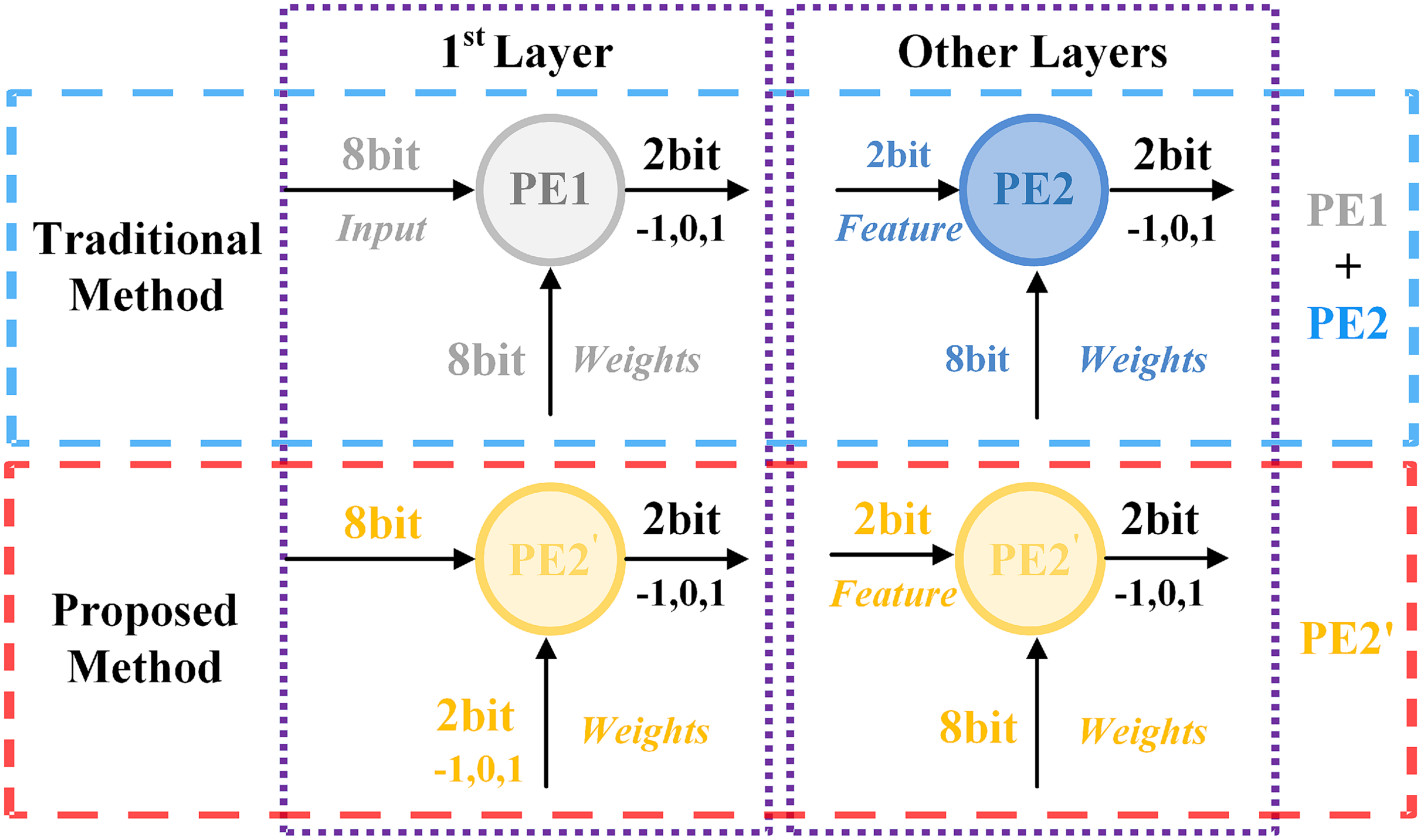
Comparison of unified PEs with traditional methods based on SNNs.

A critical flaw emerges at the hardware level, where deployment of these multi-precision networks is often architecturally “decoupled”. As illustrated in Figure 1, this paradigm necessitates designing separated processing elements (PEs) for different data bit widths (e.g., 8-bit and 2-bit). Such a decoupled PE design is fundamentally inefficient, failing to achieve full utilization of valuable on-chip computational resources.

A significant algorithm-hardware gap currently impedes the advancement of SNN deployment, presenting distinct yet interrelated challenges. At the hardware level, contemporary FPGA architectures lack unified PEs capable of supporting mixed-precision computation, thereby failing to maximize resource efficiency and computational throughput. Concurrently, at the algorithmic level, a critical need exists for hardware-aware, low-bit quantization methods that preserve model accuracy without introducing computational complexity.

This paper directly confronts this algorithm-hardware divide by proposing a novel algorithm-hardware co-design for optimizing FPGA-based computing units. Our central insight stems from identifying two distinct operational modes during SNN inference on FPGAs: (1) initial-layer computations involving weight and input pixel data, and (2) subsequent-layer computations involving weight and spike features. To reconcile these modes into a single and efficient architecture, we introduce the T8HWQ scheme. This strategy implements ternary (~2-bit) quantization for the first-layer weights and 8-bit quantization for subsequent layers. Consequently, first-layer operations are standardized as 2-bit weight × 8-bit input multiplications, while subsequent-layer operations become 8-bit weight × 2-bit spike interactions. This innovative approach unifies all network computations into a consistent 8-bit × 2-bit paradigm, enabling the design of a highly resource-efficient, unified computing architecture.

However, the aggressive first-layer quantization inevitably degrades model performance. To address this, we propose a channel-wise dual compensation strategy that recovers accuracy without increasing inference-stage computation costs. Simultaneously, building on the T8HWQ scheme, we design a unified FPGA computing architecture that supports all target operations while maximizing hardware resource reuse. In summary, the contribution of this article is as follows:

1. We propose the first algorithm-hardware co-designed T8HWQ scheme to address the heterogeneous computational patterns in SNNs. Our approach reconciles first-layer “weight × 8-bit image pixel” operations and subsequent-layer “weight × 2-bit spike” operations into a uniform 8-bit × 2-bit computation. This innovative strategy preserves model accuracy and provides a robust algorithmic basis for designing efficient, unified FPGA computing architectures.
2. To overcome the performance deficit from aggressive quantization, we introduce a novel compensation strategy with zero computational overhead at inference. This is achieved through two synergistic mechanisms: channel-wise quantization to account for feature variations and a channel-wise adaptive threshold neuron to dynamically regulate spike activation. Both are seamlessly integrated into the network weights via reparameterization, enabling our model to achieve accuracy on par with full-precision counterparts.
3. In this paper, a unified computing architecture based on the T8HWQ scheme is designed and implemented on the FPGA. By multiplexing the PE computing array, this unified approach eliminates the resource redundancy inherent in traditional decoupled PEs, achieving optimal hardware utilization without compromising computational throughput.
4. We evaluate our proposed method through both algorithm and hardware experiments. Algorithmically, on the CIFAR-10 and CIFAR-100 datasets, our approach attain near-lossless accuracy (<0.7% degradation) relative to the full-precision model in a single time step, performing competitively with other state-of-the-art (SOTA) low-bit SNNs. On the hardware front, implementation on a Xilinx XC7VX690T platform confirm that the unified computing architecture reduces lookup table (LUT) resource utilization by 20.2% compared to the traditional architecture. Compared with other advanced SNN hardware accelerators, our design delivers 6 × greater throughput than advanced SNN hardware accelerators at a comparable resource and power budget.

## 2 Related works

To deploy lightweight SNNs, quantization serves as a critical approach for compressing these networks. Unlike traditional ANNs, which utilize real-valued activations, SNNs communicate through spikes, effectively reducing the storage requirements for feature maps.

To further reduce storage space, research on quantization has focused on minimizing the bit-width of weights. All parameters are quantized to integers, including membrane potential and firing threshold (Zou et al., 2024). Furthermore, the Q-SpiNN framework is proposed for quantizing SNNs by addressing different parameters, precision levels, and rounding schemes (Putra and Shafique, 2021), reducing the bit-width to 5 bits. Additionally, spatial and temporal pruning of SNNs are implemented, decreasing the bit-width to 5 bits (Chowdhury et al., 2021). Moreover, quantization-aware training (QAT) with stacked gradient surrogation is proposed for integer-only SNNs, reducing the bit-width to 4 bits (Hasssan et al., 2024).

In recent years, to further reduce the latency of SNNs and achieve more lightweight models, researchers have further decreased the bit-width from 2 bits to 1 bit while maintaining a high latency (exceeding 5). SQUAT is proposed to enhance performance by achieving 2-bit weigh while using 25 time steps (Venkatesh et al., 2024). MINT quantizes both weights and membrane potentials to extremely low precisions in 2 bits with 8 time steps (Yin et al., 2024). Furthermore, researchers compress the weights to 1 bit (Cao et al., 2025; Eshraghian and Lu, 2022). Deng et al. (2023) propose connection pruning and weight quantization methods using ADMM optimization and activity regularization, successfully reducing the bit-width to 1, while the time steps are limited to 10 (Deng et al., 2023). Shymyrbay et al. (2023) present a framework for quantizing SNN models using a differentiable quantization function based on a linear combination of sigmoid functions achieving 1-bit weight while using 10 time steps.

Despite the significant reduction in storage space achieved by decreasing the bit width to 1-bit, performance suffers greatly. Furthermore, existing quantization studies primarily focus on minimizing bit width from the perspective of individual algorithms, overlooking the hardware costs associated with network image encoding. Given that images are typically represented in 8 bits, inconsistencies may arise between the computations of the first layer and those of subsequent layers, resulting in low resource reuse efficiency in FPGAs, as illustrated in Figure 1.

In summary, the current quantization techniques for SNNs exhibit three main characteristics: First, the use of a uniform bit width for weight quantization across all layers leads to low FPGA resource reuse efficiency, resulting in wasted computational resources. Second, as the weight bit width decreases to lower values (e.g., 2 bits), network performance declines severely, failing to achieve an optimal balance between accuracy and compression. Finally, existing quantization methods primarily focus on multiple time steps, resulting in high latency for SNNs. Consequently, these methods are unsuitable for resource-constrained and real-time applications.

To save FPGA computational resources, enhance resource efficiency, and maintain high performance under low-latency SNN conditions, we employ a ternary spike neuron with stronger information representation capabilities for SNNs. This research focuses on SNN quantization from the perspective of hardware-software co-design for FPGA implementations.

## 3 Methodology

### 3.1 The overall co-design of the quantization algorithm and hardware

This paper adopts the algorithm-hardware co-design paradigm, and at the algorithm level, the core lies in designing a quantitative strategy that is tailored to the features of the hardware. At the algorithm level, the T8HWQ quantization method is proposed to balance resource usage, processing time, and model performance. At the hardware level, FPGA design emphasizes the efficient utilization of hardware resources. Through the close collaboration of these two aspects, the aim is to build a resource-saving SNN system with both high-performance computing capabilities and low latency.

**Figure 2:**
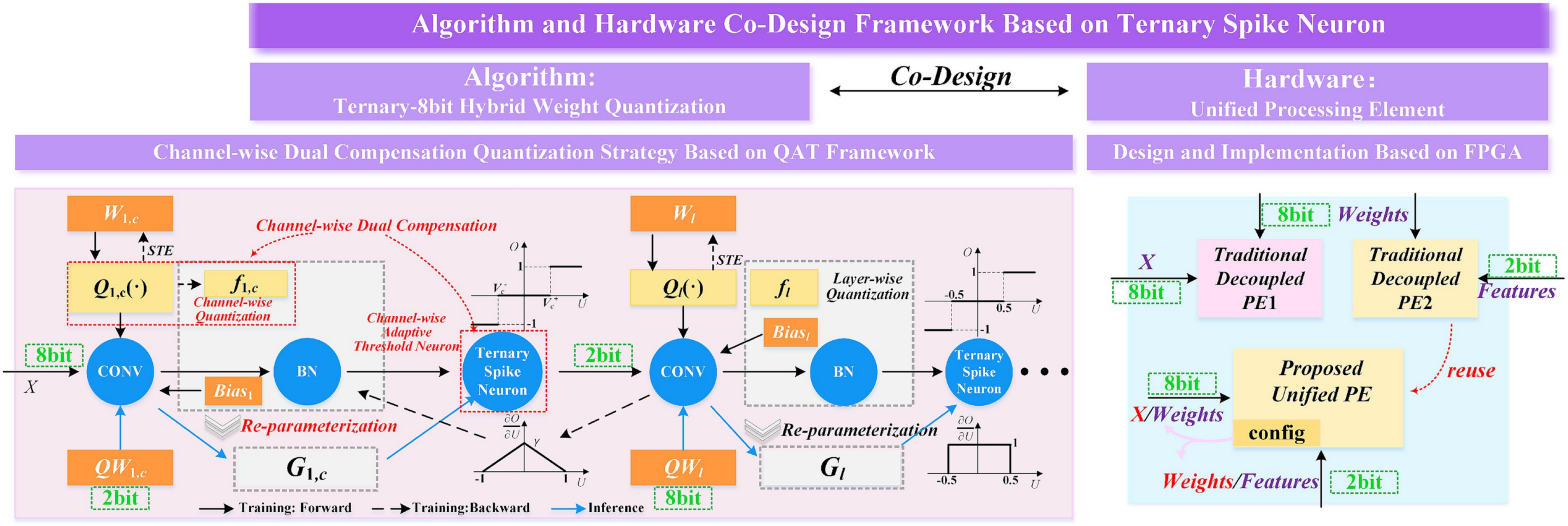
Algorithm-hardware co-design framework based on SNNs.

The algorithm-hardware co-design paradigm presented in this study includes quantization strategies at the algorithmic level, as well as design and analysis related to FPGA design, as illustrated in the Figure 2.

#### 3.1.1 Ternary-8bit hybrid weight quantization

At the algorithmic level, we have developed a hybrid bit-width quantization method T8HWQ. This method quantizes the weights of the first layer of the network into a ternary set {−1, 0, 1}. This design offers dual advantages: first, the ternary weights align with the discrete nature of neuron spike events in SNNs, allowing traditional multiplication operations to be converted into more efficient addition operations; second, ternary quantization compresses the weight storage to 2 bits, thereby reducing storage requirements. For the other layers, the data is quantized to 8 bits. Compared to traditional quantization methods, this hybrid bit-width design couples the computations across layers and simplifies the hardware design.

#### 3.1.2 The channel-wise dual compensation strategy based on QAT framework

Since the first layer serves as encoding, quantizing its weights to 2 bits inevitably leads to a certain degree of performance degradation. To address this issue, we have designed a channel-wise dual compensation strategy. This strategy effectively reduces the coding loss caused by the low bit-width quantization of the first layer by sequentially applying channel-wise quantization compensation (Krishnamoorthi, 2018) and channel-wise adaptive threshold ternary neurons compensation. Furthermore, we have constructed a quantization framework based on QAT that continuously adjusts the model to its optimal compensated state during the training phase, thereby enhancing the overall performance of the network.

#### 3.1.3 Design and implementation of a unified PE based on FPGA

From the perspective of FPGA design, the aforementioned hybrid bit-width quantization strategy offers significant advantages. In traditional hardware designs, processing data of different bit-widths typically requires distinct computational modules. However, in our design, the quantization strategy allows the FPGA to primarily handle 2-bit and 8-bit data. Based on this characteristic, we have developed a unified PE for ternary spiking neurons using FPGA. This unified PE efficiently processes both bit-widths, greatly simplifying the hardware architecture and thus saving FPGA hardware resources.

### 3.2 The channel-wise dual compensation strategy based on QAT framework

In this section, we propose a channel-wise dual compensation strategy based on QAT. We innovatively introduce adaptive threshold ternary spike neurons to channel-wise quantization, and the combination of these two compensation methods is termed the dual compensation strategy.

#### 3.2.1 Channel-wise adaptive threshold ternary spike neuron

LIF (Leaky Integrate-and-Fire) neurons are widely used as mathematical models of neuronal activity, including spike firing and the update of membrane potential. This paper adopts the ternary spike neuron (Guo et al., 2024). Compared to binary spikes, ternary spikes convey richer information. By making the positive and negative threshold parameters learnable, neurons can adaptively adjust their activation based on data and tasks, thereby enhancing overall network performance. The proposed neuron model can be represented by Equations (1) and (2):


(1) $$U_{c}^{l}(t)=\tau U_{c}^{l}(t-1)+W_{c}^{l}O_{c}^{l-1}(t)$$

(2) $$O_{c}^{l}(t)=\{\begin{array}{c} 1\\ 0\\ -1 \end{array}\begin{array}{c} \;\;\;\text{if}\;U_{c}^{l}(t)\geq V_{c}^{+}\\ \;\text{otherwise}\\ \;\;\;\text{if}\;U_{c}^{l}(t)\leq V_{c}^{-} \end{array}$$

where *τ* is a constant that describes membrane potential decaying. *U_c_^l^*(*t*) represents the membrane potential at time step *t* in the *c*-th channel of the *l*-th layer. *I* is the presynaptic input and *W_c_^l^O_c_*^*l*-1^(*t*) is the accumulation of spikes from the neurons of layer *l*-1. *W* is the weight of the neuron. *O* is the spiking output of the neuron from the previous layer. *V_c_*^+^ > 0 is the positive threshold of the *c*-th channel, *V_c_*^−^ < 0 is the negative threshold of the *c*-th channel. These two learnable thresholds enable neurons to find appropriate activation thresholds for different channels. We employ a soft reset mechanism represented by Equation (3):


(3) $$U_{c}^{l}(t)=\{\begin{array}{c} U_{c}^{l}(t)-V_{c}^{+},\\ U_{c}^{l}(t),\\ U_{c}^{l}(t)-V_{c}^{-}, \end{array}\begin{array}{c} \text{if}\;O_{c}^{l}(t)=+1\\ \text{if}\;O_{c}^{l}(t)=0\\ \text{if}\;O_{c}^{l}(t)=-1 \end{array}$$

SNNs output spike sequences instead of continuous numerical values, presenting challenges for direct application of traditional backpropagation algorithms. To address this, researchers have utilized surrogate gradients (Neftci et al., 2019) to train SNNs using backpropagation methods. During forward propagation, a non-differentiable spiking function is employed, while backpropagation utilizes a continuously differentiable surrogate function for gradient computation. The surrogate gradient of membrane potential is defined by Equation (4):


(4) $$\frac{\partial O}{\partial U}=\{\begin{array}{cc} 1 & \text{if}-1\leq U\leq 1\\ 0 & \text{otherwise} \end{array}$$

The surrogate gradient of the threshold is defined by Equation (5):


(5) H(U,V)=γ⋅max(0,1−∣U−V∣)

The gradient of the weights is defined by Equations (6–8):


(6) $$\frac{\partial L}{\partial W^{l}}=\frac{\partial L}{\partial O^{l}}\cdot \frac{\partial O^{l}}{\partial U^{l}}\cdot \frac{\partial U^{l}}{\partial W^{l}}$$

(7) $$\frac{\partial O^{l}}{\partial U^{l}}=H(U,0)=\gamma \cdot \max(0,1-|U|)$$

(8) $$\frac{\partial U^{l}}{\partial W^{l}}=\{\begin{array}{c} X\\ O^{l-1} \end{array}\begin{array}{c} \;\;\text{if}\;l=1\\ \;\;\text{if}\;l>1 \end{array}$$

The gradient of the positive threshold is defined by Equation (9):


(9) $$\frac{\partial L}{\partial V^{+}}=\frac{\partial L}{\partial O}\cdot \frac{\partial O}{\partial V^{+}}=\frac{\partial L}{\partial O}\cdot (-H(U,V^{+}))$$

The gradient of the negative threshold is defined by Equation (10):


(10) $$\frac{\partial L}{\partial V^{-}}=\frac{\partial L}{\partial O}\cdot \frac{\partial O}{\partial V^{-}}=\frac{\partial L}{\partial O}\cdot H(U,V^{-})$$

#### 3.2.2 Quantization scheme and reparameterization technique

##### 3.2.2.1 First layer quantization scheme

In this paper, we employ 2-bit channel-wise quantization for the first layer weights and 8-bit layer-wise quantization for the remaining layers. Compared to full-precision networks, ternary weight quantization reduces the precision to three discrete values, which is defined by Equation (11):


(11) $$Q(W)=\{\begin{array}{ll} +1, & \text{if}\;W\geq \theta_{1}\\ 0, & \text{if}\;\theta_{2}<W<\theta_{1}\\ -1, & \text{if}\;W\leq \theta_{2} \end{array}$$

where $\theta_{1}$ and $\theta_{2}$ are the quantization thresholds, with $\theta_{1}>\theta_{2}$ . We adopt a symmetric form of the quantization thresholds, that is, *θ*_1_ = 0.5 and *θ*_2_ = −0.5. Then the channel-wise quantization function can be simplified in Equation (12):


(12) $$Q_{l,c}(W_{l,c})=\{\begin{array}{ll} \mathrm{sign}(W_{l,c}) & \text{if}\;\frac{|W_{l,c}|}{f_{l,c}}\geq \theta \\ 0 & \text{otherwise} \end{array}$$

where *l* indicates the index of the network layer, and *c* represents the index of the output channel of the layer. *f* is the channel scaling factor, which is related to the number of channels in the convolutional kernel. For the convolutional kernel weights $W_{l,c}\in {\mathbb{R}}^{C_{l,\text{out}}\times C_{l,\text{in}}\times K\times K}$ , where $C_{l,\text{out}}$ is the number of output channels, $C_{l,\text{in}}$ is the number of input channels, *K* is the kernel size. If *N* is the number of layers in the network, then $l\in \{1,2,\ldots ,N\},c\in \{1,2,\ldots ,C_{l,\text{out}}\}$ .

The channel weight matrix can be defined as $W^{\left(c\right)}\in {\mathbb{R}}^{C_{\text{in}}\times K\times K}$ . To exclude the influence of outliers, we define the first-layer channel scaling factor *f*, as given in Equations (13–15):


(13) $$q_{\max}^{\left(c\right)}=\inf\{w\in \mathbb{R}|P(W^{\left(c\right)}\leq w)\geq 0.99\}$$

(14) $$q_{\min}^{\left(c\right)}=\sup\{w\in \mathbb{R}|P(W^{\left(c\right)}\geq w)\geq 0.01\}$$

(15) $$f_{1,c}=\max(|q_{\max}^{\left(c\right)}|,|q_{\min}^{\left(c\right)}|)\;\forall c\in [1,C_{l,\text{out}}]$$

where inf represents the infimum and sup represents the supremum. $q_{\max}^{\left(c\right)}$ is a threshold indicating that 99% of the channel weight values are less than or equal to this value, while $q_{\min}^{\left(c\right)}$ is a threshold indicating that 1% of the channel weight values are greater than or equal to this value. Due to the non-differentiability of the round and clip, we employ the Straight-Through Estimator (STE) for backpropagation to update the weights (Bengio et al., 2013), as shown in Equation 16.


(16) $$\frac{\partial Q_{1,c}}{\partial W_{1,c}}=\{\begin{array}{cc} 1 & \text{if}\;|\frac{W_{1,c}\cdot S}{f_{1,c}}|<S\\ 0 & \text{otherwise} \end{array}$$

##### 3.2.2.2 The other layers quantization scheme

All subsequent layers employ 8-bit layer-wise weight quantization, with the quantization function defined by Equations (17) and (18):


(17) $$Q_{l}=\frac{f_{l}}{2^{b-1}-1}\cdot \text{round}((2^{b-1}-1)\cdot \text{clip}(\frac{W_{l}}{f_{l}},-1,1))\forall l\in [2,N]$$

(18) $$\frac{\partial Q_{l}}{\partial W_{l}}=\{\begin{array}{cc} 1 & \text{if}\;|W_{l}|<f_{l}\\ 0 & \text{otherwise} \end{array}$$

where *W_l_* represents the 32-bit floating-point weights and *b* is the integer bit width set to 8, and thus *S* is 127. Our proposed quantization framework is illustrated in Algorithm 1.

**ALGORITHM 1:**
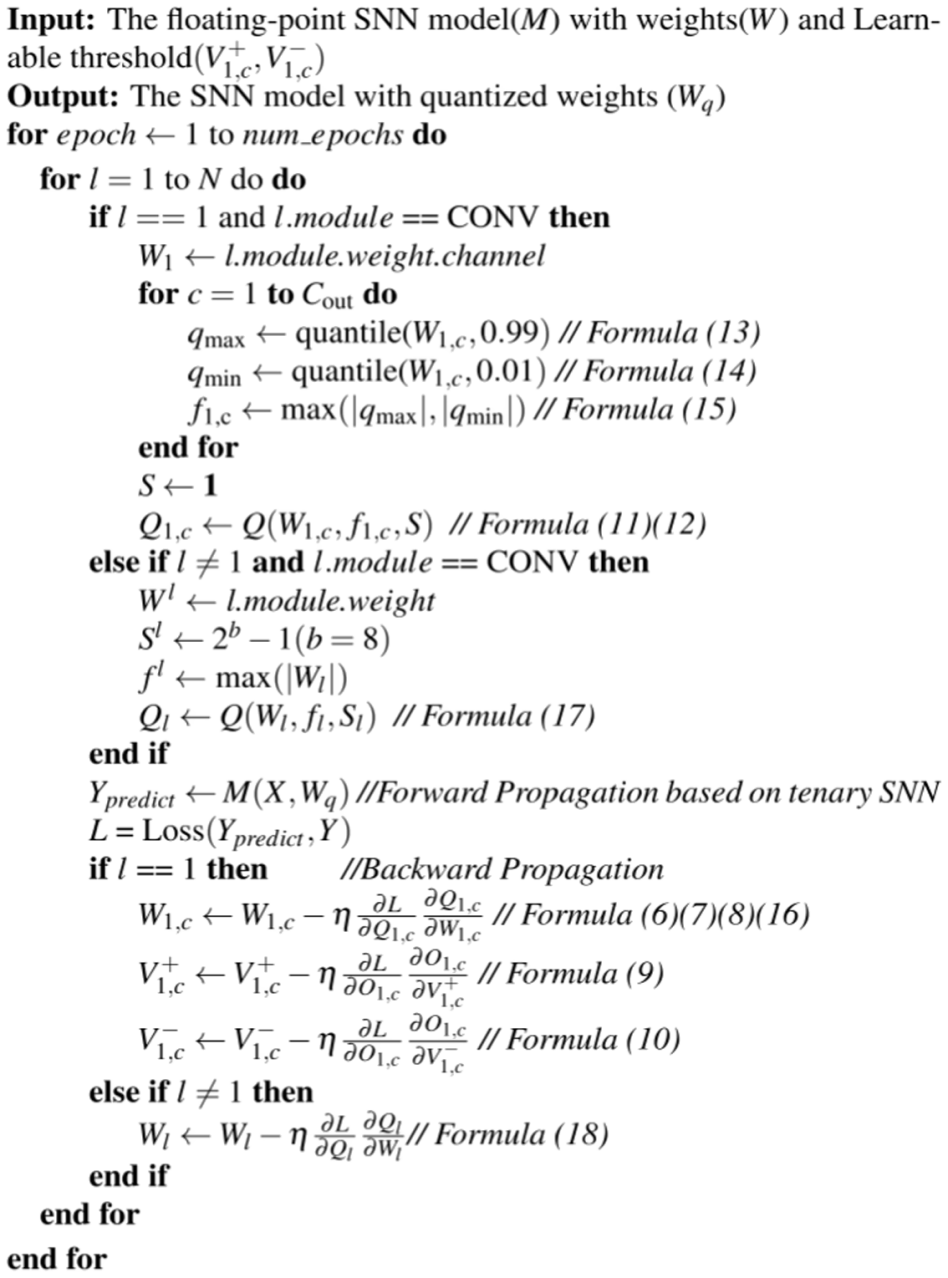
Proposed quantization framework.

##### 3.2.2.3 Reparameterization technique

Channel-wise quantization is introduced at the first layer, increasing the training overhead. Nonetheless, we use a reparameterization technique that eliminates additional computation during the inference phase. The quantized weights pass through the convolutional layer and BN (Batch Normalization) layer, and the results are:


(19) $$\begin{array}{l} G_{1,c}=\frac{\gamma_{c}\cdot f_{1,c}}{\sigma_{c}}\cdot \text{float}(X*QW_{1,c})+\\ \;\beta_{c}-\frac{\gamma_{c}\mu_{c}}{\sigma_{c}}=\hat{a_{c}}\cdot \text{float}(X*QW_{1,c})+{\hat{b}}_{c} \end{array}$$

where *σ* is the variance, *μ* is the mean, and *γ* and *β* are two learnable parameters. *X* represents the input image of the first layer, *QW*_1*,c*_ denotes the quantized ternary weights of the first layer. The term “float” indicates the conversion from fixed-point to floating-point representation. The data width of *X* is 8-bit, while *QW*_1*,c*_ is quantized into 2-bit, with all other parameters being 32-bit floating-point. The convolutional bias is omitted in this implementation and thus excluded from the Equation 19.

As shown in Equation 19, $\hat{a_{c}}$ and $\hat{b_{c}}$ correspond to the output channel of the first layer, and the BN calculation can be converted to a single floating-point multiplication and a single floating-point addition. The computational operations of our dual compensation strategy match those of both layer-wise and channel-wise quantization, as summarized in Table 1. However, channel-wise dual compensation requires additional 4 × (*C_out_* − 1) × 32-bit storage to maintain the floating-point multiply-add results and positive/negative thresholds. In short, the dual compensation boosts performance without extra computation, needing only more storage.

**Table 1:** Comparison of floating-point operations and memory overhead between dual-compensation strategy and traditional quantization methods (*C_out_* denotes output channels).

Methods	Floating-point multiplication	Floating-point addition	Floating-point storage (32bit)
Layer-wise quantization	*C_out_*	*C_out_*	4
Channel-wise quantization	*C_out_*	*C_out_*	2*C_out_* + 2
Proposed	*C_out_*	*C_out_*	4*C_out_*

### 3.3 Design of the unified PE based on FPGA

#### 3.3.1 Parallelism of unified PEs

**Figure 3:**
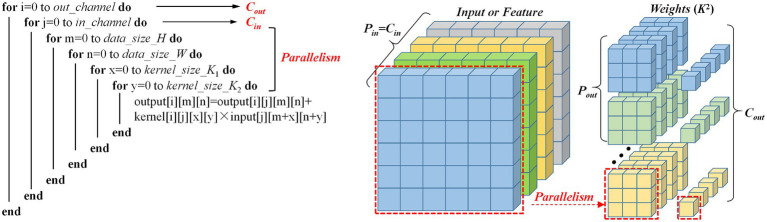
Parallel computing architecture and pseudocode implementation of multi-channel convolution.

Convolutional layers serve as fundamental feature extraction modules in modern deep learning models. A standard convolutional layer computes an output feature map Y (*C_out_* × *H′* × *W′*) by convolving an input feature map X (*C_in_* × *H* × *W*) with a filters W (*C_out_* × *C_in_* × *K* × *K*), formally expressed as given in Equation (20):


(20) $$Y(i,m,n)=b\_i+\sum_{j=0}^{C_{\text{in}}-1}\sum_{x=0}^{K-1}\sum_{y=0}^{K-1}W(i,j,x,y)\times X(j,m+x,n+y)$$

where *i* and *j* index the output and input channels respectively, (*m*,*n*) represent the spatial coordinates in the output feature map, *K* is the kernel size, and *b*_*i* represents the bias term. The above operation can be broken down into a cyclic structure as shown in the Figure 3. The diagram shows the calculation process of the standard convolutional layer and its parallelism. One parallelism represents the sliding the 2D weight kernel across the 2D input feature map. The key parameters and parallelism dimensions are defined as follows:

Input Channels (*C_in_*): This parameter defines the input feature map depth, representing the number of stacked 2D feature maps. As shown in the Figure 3, the “Input or Feature” cube’s depth dimension corresponds to *C_in_*; in the pseudocode, *C_in_* determines the layer 2 loop boundary, requiring full traversal of all input channels during each output channel computation.

Output Channels (*C_out_*): This parameter represents the number of filters in the convolutional layer and determines the depth dimension of the output feature map.

Input Channel Parallelism (*P_in_*): This parameter defines the parallel processing capacity across input channels when computing a single output feature map. When *P_in_* = *C_in_*, as shown in Figure 3, the convolutional filter simultaneously processes all *C_in_* channels and completes accumulation in one computational step. From a hardware implementation perspective, this requires sufficient compute units to process data from all input channels in parallel, thereby speeding up the computation of a single output channel. In our work, the value of the *P_in_* is 512.

Output Channel Parallelism (*P_out_*): As another parallel computing dimension, it refers to the number of output channels that can be computed at the same time. Since each output channel’s computation is independent (e.g., the *i*-th output channel does not depend on the results of the *i* + 1 channel), distinct filters can be applied in parallel. As shown in the Figure 3, when a *P_out_* filter is applied to the input feature map in parallel, the Pout output channels can be computed simultaneously. In our implementation, *P_out_* is set to 1.

Since the hidden layer’s feature map uses 2-bit ternary values {−1, 0, 1}, convolution multiplications simplify to additions. The first-layer weights are reduced to 2 bits via ternary quantization. This approach retains the input image at 8-bit precision but enables computing unit reuse through configurable operations.

#### 3.3.2 Design of the unified PE architecture based on FPGA

**Figure 4:**
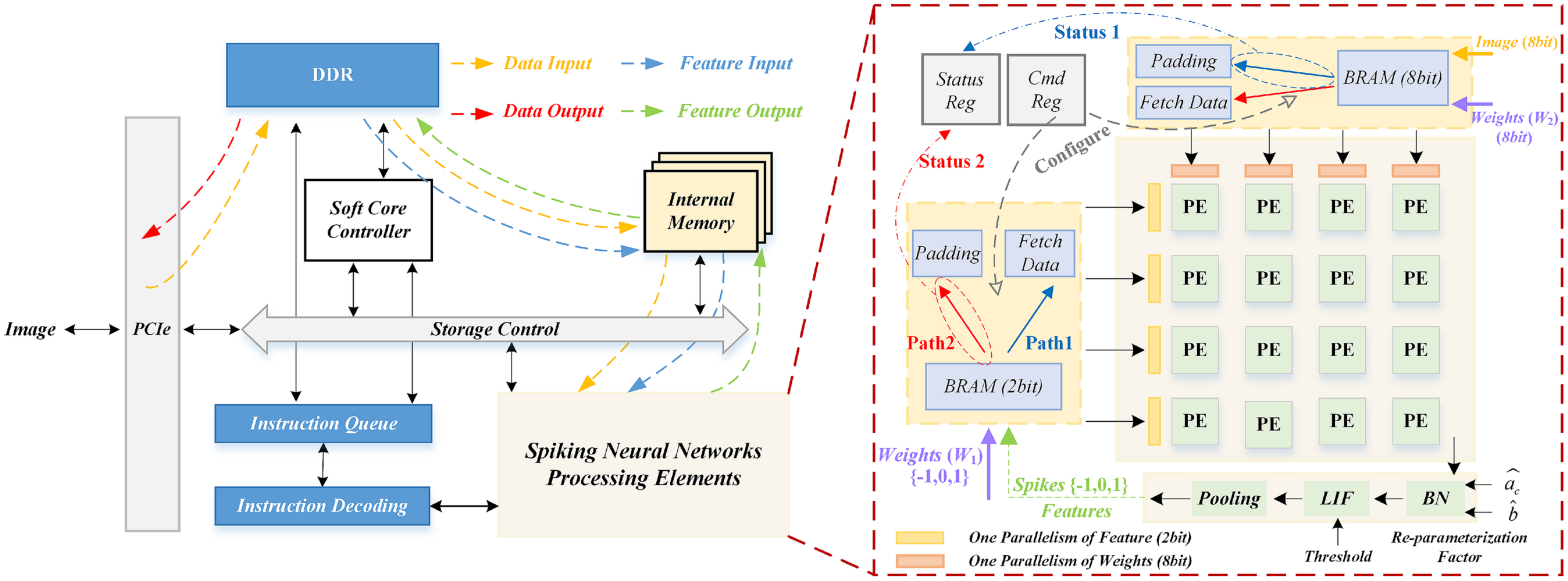
FPGA-based hardware accelerator architecture and overall system for SNNs.

Based on the above analysis, we implement the unified computing architecture on the FPGA, as shown in Figure 4. Our SNN acceleration system comprises three key components: data storage, control path and computing core.

##### 3.3.2.1 Data storage

**External Interface:** The system interfaces with the host computer via a Peripheral Component Interconnect Express (PCIe) bus, which serves as the primary channel for raw image data transmission. Double Data Rate SDRAM (DDR) provides high-capacity storage for network weights and intermediate feature maps. The yellow arrows (data input) and red arrows (Data Output) in the diagram indicate the data flow between the DDR and external interfaces.

**Internal Memory:** The architecture incorporates on-chip BRAM serving as a data cache. This memory temporarily stores layer-specific weights and feature maps (blue feature input arrows) fetched from DDR, while buffering output feature maps (feature output arrows) processed by PEs.

**Storage Control:** This is a scheduling module that manages the transfer of data between the DDR, BRAM, and SNN processing cores. Through Direct Memory Access (DMA) operations, it ensures timely data delivery to the PE array according to execution requirements.

##### 3.3.2.2 Control path

**Soft core controller:** The system employs a MicroBlaze processor to orchestrate the inference pipeline, managing network weight loading, PE configuration timing, and coordination with both the storage controller and instruction queue for flexible hardware control.

**Instruction queue and decoding:** The controller dispatches predefined instructions to the instruction queue. A dedicated decoding module sequentially decodes these instructions into precise control signals for both the SNN processing core and storage controller. This architecture ensures programmability while supporting diverse SNN architectures.

##### 3.3.2.3 Computing core

The unified computing architecture comprises three key components: PE array, dual data paths, configuration and state control, as shown in Figure 4.

**PE array:** It implements convolution calculations of inputs and weights. It here supports 512 parallelism.

**Dual data paths:** These two data paths support 8-bit image input and 2-bit feature input, respectively. In the respective data pathways, the data is fed into the PE array by the necessary pad filling or directly reading (Fetch Data and Padding).

**Configuration and state control:** It consists of command registers (Cmd Reg) and status registers (Status Reg). The Cmd Reg receives configuration instructions from the instruction decoder. The Configure signal determines which data path (8-bit BRAM path or 2-bit BRAM path) to enable based on whether the first or subsequent layer is currently being calculated, and directs the corresponding data to the PE array. The Status Reg collects status signals (Status 1/2) from the data path, such as data readiness, computation completion. These statuses are fed back to the soft core controller. This configuration logic is summarized in Table 2 and the overall workflow utilizing these configurations is detailed in Table 3.

**Table 2:** Configuration logic for dual data path selection.

Computation stage	Configure signal	Enabled data path	Status signal	Input type	Weight bitwidth
First layer	Select Path 1	8-bit BRAM Path	1	8-bit Image	2-bit
Subsequent layers	Select Path 2	2-bit BRAM Path	2	2-bit Feature	8-bit

**Table 3:** Workflow and state transition mechanism of unified PE.

Step	Stage 1: first-layer computation	Stage 2: subsequent-layer computation
1. Configuration	Trigger condition: To process the first layer of the network. Operation: Cmd Reg issues instructions to switch to Path 1.	Trigger condition: To process the second and subsequent layers of the network. Operation: Cmd Reg issues instructions to switch to Path 2.
2. Data Flow	Data source: Original image. Storage: 8-bit image data loaded into 8-bit BRAM. Transmission: Data passes through the Padding module and enters PE.	Data source: Spike feature maps. Storage: 2-bit feature data loaded into 2-bit BRAM. Transmission: Data passes through the Padding module and enters PE.
3. Weight Flow	Weight source: First-layer weights (*W*_1_). Transmission: 2-bit weights pass through the Fetch Data module and enters PE.	Weight source: Subsequent-layer weights (*W*_2_). Transmission: 8-bit weights pass through the Fetch Data module and enters PE.
4. Core Computation	The PE array performs convolution: 8-bit images × 2-bit weights.	The PE array performs convolution: 2-bit features × 8-bit weights.

After the PE array completes the convolution, the results undergo post-processing including BN and LIF modules to enable neuronal dynamics of the SNN and activate spikes.

**BN module:** The convolutional outputs are normalized before entering the neuron model. In hardware implementations, the parameters of the BN, *γ* and *β*, are typically fused with convolutional weights. Reparameterization factor $\hat{a_{c}}$ and $\hat{b_{c}}$ simplifying the BN operation to one multiplication and one addition.

**LIF module:** This module receives the value after BN and updates the internal membrane potential according to LIF neural dynamics. The updated membrane potential is compared to a configurable threshold: if the membrane potential exceeds the positive threshold, the neuron emits a spike (+1); if the membrane potential exceeds the negative threshold, the neuron will emits a spike (−1); Otherwise, the spike is not activated (0). In this work the time step is set to 1, the membrane potential is not further updated.

**Pooling module:** The maximum pooling operation is performed on the spike feature map, reducing spatial dimensions, expanding the receptive field, and improving the model’s translation invariance.

**Final output:** The results of pooling (the new spike feature map ∈ {−1, 0, 1}) will be written back to the internal memory as input to the next layer, thus completing a full computation cycle.

In summary, through the innovative configurable dual data path design, the unified PE array can support computing of the first layer (8-bit input) and the subsequent layer (2-bit input) on the same computing arrays. This innovation ensures full reuse of computational resources across all network layers, significantly enhancing hardware utilization.

## 4 Experiments and discussion

### 4.1 Datasets and evaluation metrics

#### 4.1.1 Dataset

In this study, we utilize CIFAR10 andCIFAR100 datasets. The CIFAR10 dataset (Krizhevsky and Hinton, 2009) comprises 60,000 color images measuring 32 × 32 pixels, distributed across 10 distinct classes. It consists of 50,000 samples for training and an additional 10,000 samples for validation. CIFAR-100 is more challenging. It has 100 classes containing 600 images each.

#### 4.1.2 Evaluation metrics

For performance evaluation, we utilize overall accuracy to assess classification performance. Furthermore, the signal-to-noise ratio (SNR) is used to evaluate the robustness of the quantized model.

### 4.2 Implementation details

#### 4.2.1 Data preprocessing and networks

For CIFAR10 and CIFAR100 dataset, during training, we apply standard data augmentation techniques, which include adding a 4-pixel padding on each side, performing random 32 × 32 cropping, and applying random horizontal flipping. However, during validation, the original images are utilized without these techniques. All the images are normalized to achieve a zero mean and unit variance. We employ VGG16, VGG11 (Simonyan and Zisserman, 2015) and ResNet19 (He et al., 2016) for validation on CIFAR10/100datasets.

#### 4.2.2 Hyperparameters setting

During training, we employ a cross-entropy loss function with stochastic gradient descent optimization which incorporates weight decay (0.0005) and momentum (0.9) parameters. The full precision SNNs are trained for 300 epochs on the all datasets. The learning rate is 0.1 for VGG architectures and 0.01 for ResNet network, with a batch size of 256. We adopt a cosine learning rate decay schedule during training. During quantization stage, the AdamW optimizer is used with a weight decay of 0.01. The leaky factor 𝜏 is fixed at 1 and the firing threshold 𝜃 for ternary spike neurons is initialized at 0.5. The time step is set to 1 for all experiments. We utilize Python 3.10 and PyTorch 1.12 software and two NVIDIA A6000 Graphical Processing Units (GPUs). The operating system is Ubuntu 18.04.

#### 4.2.3 Implementation details of hardware

For the FPGA implementation, we use Verilog and Vivado 2020.2 to design the architecture. The power consumption data comes from the power report provided by the software. The unified PE is deployed on the Xilinx Virtex-7 XC7V690T FPGA operating at 100 MHz clock frequency. We adopt the row-stationary strategy used in works like Eyeriss (Chen et al., 2016), where kernel-sized rows of the feature map are stored in on-chip cache at the dataflow level. For example, a 3 × 3 convolution requires caching 3 rows of input data. For each new row processed, the cache updates to present a continuous convolution dataflow to the PEs. This maximizes data reuse and computational efficiency.

### 4.3 Algorithm performance evaluation

#### 4.3.1 Performance comparison with advanced methods

To evaluate the effectiveness of our method, we conduct comparative experiments with existing quantized SNN approaches on the CIFAR-10 dataset. The results are summarized in Table 4, organized into four cases based on quantization bit-widths for the first layer weights (*W*_1_) and subsequent layers weights (*W*_2_): 32/32, 8/8, 2/8, and 2/2. The notation “*a*/*b*” indicates *a*-bit quantization for *W*_1_ and *b*-bit quantization for *W*_2_.

**Table 4:** Comparison of the performance of VGG16 and ResNet19 with other SOTA methods on CIFAR10.

Dataset	Method	Model	Precision (*W*_1_/*W*_2_)	Time step	Accuracy (%)
CIFAR10	Yin et al. (2024)	Vgg16	8/8	8	90.72
	Yin et al. (2024)	Vgg16	32/32	8	91.15
	Yin et al. (2024)	ResNet19	32/32	8	91.29
	Yin et al. (2024)	ResNet19	8/8	8	91.36
	Zhou et al. (2021)	VGG16	2/2	-	90.93
	Yoo and Jeong (2023)	VGG16	2/2	32	**91.66**
	Xu et al. (2023)	VGG16	32/32	4	91.05
	This work	ResNet19	32/32	1	91.95
	This work	ResNet19	2/8	**1**	**91.79**
	This work	VGG16	32/32	1	91.93
	This work	VGG16	2/8	**1**	91.55

Bold values indicate superior performance.

The proposed quantization method achieves low latency and high performance in SNNs. As shown in Table 4, method (Yoo and Jeong, 2023) require 32 time steps to reach 91.66% accuracy, whereas our approach attains 91.55% accuracy in single time step, reducing latency by 32 × . By optimizing the quantization strategy, our method minimizes quantization loss while maintaining competitive network performance. Experiments on VGG16 and ResNet19 architectures demonstrate accuracies of 91.55% and 91.79%, respectively, outperforming prior results reported by Yin et al. (2024) at 90.72% and 91.36%.

To systematically evaluate the effectiveness of our method, we conduct comparative experiments with other SOTA approaches on the CIFAR-100 dataset, as summarized in Table 5.

**Table 5:** Comparison of the performance of VGG11 and ResNet19 with other SOTA methods on CIFAR100.

Dataset	Method	Model	Precision (*W*_1_/*W*_2_)	Time step	Accuracy (%)
CIFAR100	Zou et al. (2024)	VGG11	32/32	–	67.40
	Zou et al. (2024)	VGG11	2/2	4	54.27
	Wang et al. (2025)	VGG16	32/32	-	77.22
	Wang et al. (2025)	VGG16	32/32	2	64.89
	Gao et al. (2023)	VGG16	8/8	8	66.32
	Hasssan et al. (2024)	ResNet19	32/32	2	72.78
	Hasssan et al. (2024)	ResNet19	4/4	2	71.87
	This work	ResNet19	32/32	1	72.88
	This work	ResNet19	2/8	1	**72.23**
	This work	VGG11	32/32	1	67.84
	This work	VGG11	2/8	1	**67.38**

Our method demonstrates clear advantages in inference efficiency, which is especially important in SNNs where the number of time steps (T) directly affects system latency and computational overhead. Experimental results show that the proposed method achieves single-time step inference across multiple architectures, such as ResNet19 and VGG11. In contrast, methods from Zou et al. (2024) (T = 2), Hasssan et al. (2024) (T = 2) and Gao et al. (2023) (T = 8) all require multiple time steps to complete inference. This single-time-step capability makes our approach particularly suitable for latency-sensitive edge computing applications, such as autonomous driving and industrial inspection, where rapid inference is crucial.

In addition, our method maintains high model performance within a single time step. For instance, when quantizing ResNet19 from 32-bit full precision to 2/8-bit, the accuracy only decreases slightly from 72.88 to 72.23%, with a loss of 0.65%. Similarly, On VGG11 declines marginally from 67.84 to 67.38%, with a loss of 0.46%. Compared with the full-precision VGG16 (64.89%) reported by Wang et al. (2025), our approach achieves an improvement of 2.49%. Additionally, compared with the 2-bit quantified VGG11 (54.27%) reported by Zou et al. (2024), our method’s performance increases by 13.11%. While the 4-bit quantization scheme (Hasssan et al., 2024) achieves 71.87% accuracy in two time steps, our method achieves a comparable performance in one time step. These results demonstrate that our quantization strategy enables high-performance model compression without increasing the number of time steps, providing efficient algorithmic support for hardware-accelerated unified computing architectures.

#### 4.3.2 Hardware-oriented performance trade-off analysis: SNN vs. ANN

To thoroughly assess the proposed method’s effectiveness, this section compares our T8HWQ SNN with the W2A8 ANN, a ternary weight network (TWN) (Liu et al., 2023) with 8-bit activation quantization. The comparison focuses on accuracy and storage overhead, as shown in Table 6.

**Table 6:** Comparison of the proposed SNN and ANN on performance and storage overhead.

Dataset	Method	Model	*W*_1_/*W*_2_/*W_A_*	Weight (MB)	Feature map (KB)		Accuracy (%)
					32 × 32	640 × 640	
CIAFR100	TWN	VGG11(ANN)	32/32/32	35.88	10.66	4262.50	**69.52**
	TWN	VGG11(ANN)	2/2/8	**2.24**	2.66	1065.62	66.90
	This work	VGG11(SNN)	32/32/2	35.88	0.67	266.41	67.84
	This work	VGG11(SNN)	2/8/2	8.97	**0.67**	**266.41**	**67.38**
	TWN	Resnet19(ANN)	32/32/32	47.64	36.25	14,500	**74.21**
	TWN	Resnet19(ANN)	2/2/8	**2.98**	9.06	3625.00	71.45
	This work	Resnet19(SNN)	32/32/2	47.64	2.26	906.25	72.88
	This work	Resnet19(SNN)	2/8/2	11.91	**2.26**	**906.25**	**72.23**

Bold values indicate superior performance.

While the W2A8 ANN can employ the same unified computing architecture as ours, experimental results demonstrate that our method offers accuracy benefits. On CIFAR-100, with a single time step, our SNN achieves 67.38 and 72.23% accuracy on VGG11 and ResNet19, respectively, surpassing TWN’s 66.90 and 71.45%. This indicates that, under ultra-low latency inference constraints, our SNN can still outperform the W2A8 ANN using identical hardware, revealing its higher performance potential.

To improve accuracy, our model balances weight storage against feature map storage. As shown in Table 6, ResNet19’s weight storage is 11.91 MB with our method, compared to 2.98 MB for TWN. Nevertheless, our approach reduces feature map storage and processing overhead. Since our activation values are only 2 bits, feature map storage decreases by approximately 75%, for example, from 9.06 KB to 2.26 KB in ResNet19. In edge computing chip design, the main performance constraint lies not only in computation but also in data movement. Weight parameters are read once during inference and stored in off-chip DRAM. Conversely, feature maps require frequent read/write operations and must reside in on-chip SRAM to enable low-latency, high-bandwidth data transfer and lower power consumption.

This challenge is particularly prominent in ResNet networks using residual connections: the shallow network’s output feature map must be retained on-chip before being added to the deeper feature map after multiple convolutional layers. When on-chip SRAM capacity is insufficient, these feature maps are offloaded to external DRAM and reloaded, incurring substantial latency and power consumption (Bhati et al., 2016). Therefore, drastically reducing feature map storage via quantizing activation values to very low bit-widths is a vital strategy to mitigate this challenge and enable efficient hardware acceleration.

This advantage becomes even more pronounced with high-resolution inputs. As shown in the table, the feature map size roughly scales with the square of the input dimension (*N*^2^). When the input size increases to 640 × 640, the ANN’s feature map storage rises sharply to 3,625 KB, whereas our method requires only 906 KB, achieving a fourfold reduction. This scalability demonstrates our approach’s strong potential for large-scale data processing, such as high-resolution remote sensing image analysis (Maggiori et al., 2017).

In summary, our single-step SNN model outperforms the W2A8 ANN in accuracy with the same computing architecture. Although it reduces weight storage, this trade-off enables improvements in feature map memory, data movement efficiency, and power consumption. This hardware-software co-design offers a promising approach for processing large-scale data on edge devices.

#### 4.3.3 Feature analysis

**Figure 5:**
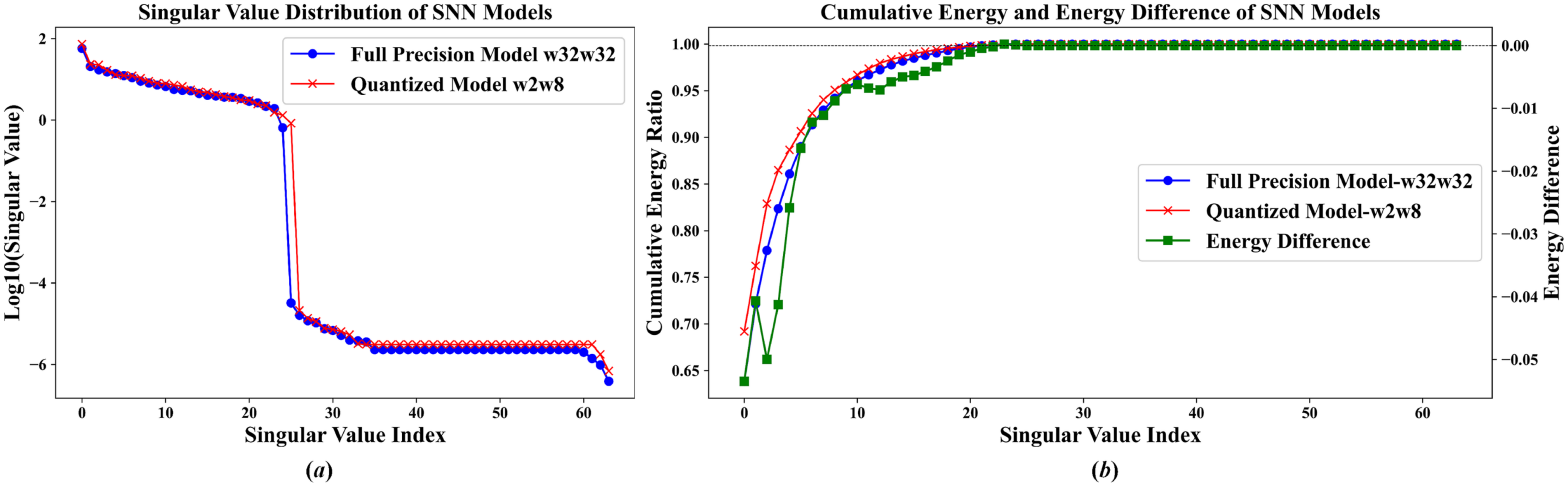
Feature analysis of VGG16 on CIFAR10 dataset **(a)** singular value distribution comparison between full-precision (w32w32) and quantized (w2w8) models **(b)** cumulative energy ratio and energy difference.

**Figure 6:**
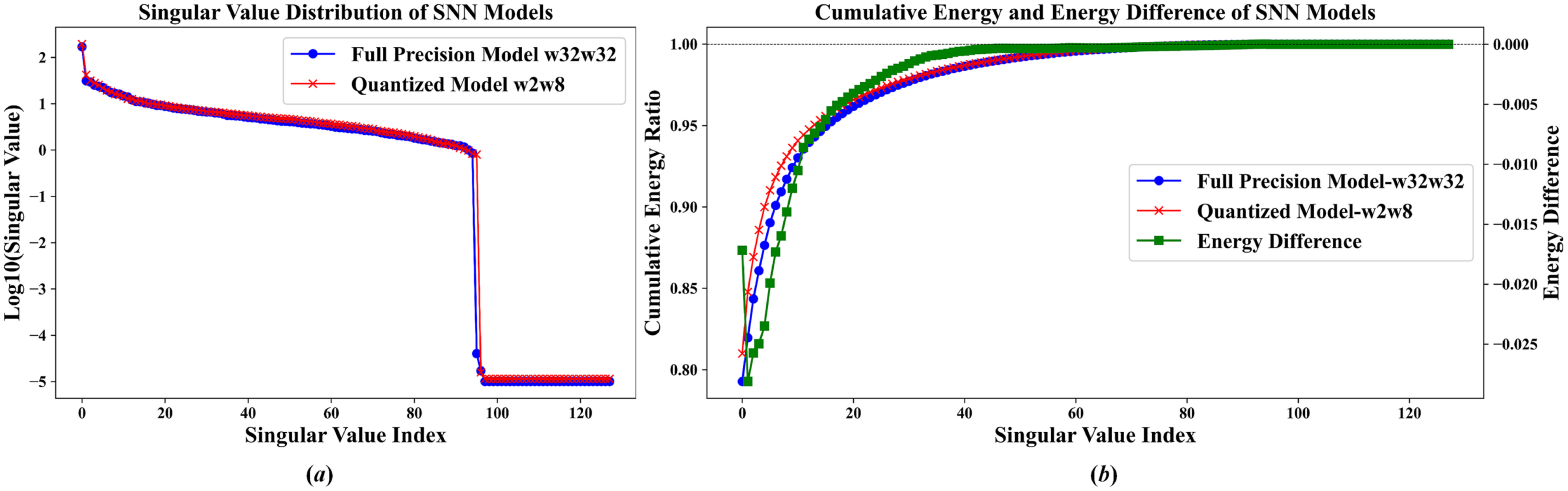
Feature analysis of ResNet19 on CIFAR10 dataset **(a)** singular value distribution comparison between full-precision (w32w32) and quantized (w2w8) models **(b)** cumulative energy ratio and energy difference.

To systematically explore the information retention ability of the first layer in the quantized model, we employ singular value decomposition (SVD) on the channel characteristic map of this layer. This analysis involves examining the singular values and their cumulative energy distribution, as shown in Figures 5, 6.

The results shown in Figures 5A, 6A indicate that the proposed quantization scheme successfully retains the key information of the original full-precision model while realizing model compression. Among them, the blue curve representing the full-precision model and the red curve representing the quantization model nearly overlap, demonstrating that their singular value distributions are highly similar. Specifically, as shown in Figure 5 the quantized SNN model closely matches the full-precision model’s singular values within the first 25 maximum singular values. Similarly, in Figure 6, the quantized model exhibits near-identical characteristics for the singular values up to the 95th index. These observations confirm that the quantized model successfully maintains the core information and essential functions of the original full-precision network.

The results displayed in Figures 5B, 6B demonstrate that the energy distribution of the quantized model closely aligns with that of the full-precision model. Specifically, in Figure 5B, the top 10 singular values already capture more than 90% of the total energy contribution, with the energy difference within these singular values fluctuating by no more than 0.05. Beyond the 10th singular value, the energy difference diminishes further, remaining below 0.01. Similarly, Figure 6B shows that the first 20 singular values account for approximately 95% of the energy, and for singular values with indices greater than 20, the energy difference between the quantized and full-precision models is less than 0.005. These findings indicate that the overall energy difference between the full-precision and quantized models is minimal, suggesting that the quantization process effectively preserves the energy distribution of the original model.

Additionally, Figure 5B illustrates the impact of quantization noise on the model’s performance. Specifically, when the singular value index exceeds 25, the red curve representing the quantized model remains above the blue curve of the full-precision model. For these higher-index singular values, which are inherently small, the energy introduced by quantization noise becomes significantly larger than that of the original signal. As a result, the singular values at these locations no longer accurately reflect the fine details of the original model. In particular, at the 26th index, the singular value of the quantized model is markedly larger than that of the full-precision model, indicating that the delicate information contained in the full-precision model has been overwhelmed by noise. This phenomenon suggests that excessive quantization noise at these higher indices can potentially degrade the overall model accuracy by obscuring subtle but important features.

In summary, the quantization approach proposed in this study exhibits both negative and positive effects. On the negative side, quantizing the first layer of the model to 2 bits inherently introduces quantization noise, which can lead to a decline in the overall network performance. Conversely, the analysis based on singular values and energy distributions demonstrates that the proposed method effectively preserves the core functions and essential information of the original model. By accurately maintaining key singular values and the associated energy distributions, the approach ensures that the fundamental capabilities of the network are largely retained. Consequently, this compression strategy successfully reduces model size and complexity while preserving the critical information of the full-precision model, achieving a balance between efficiency and performance.

### 4.4 Robustness evaluation

**Figure 7:**
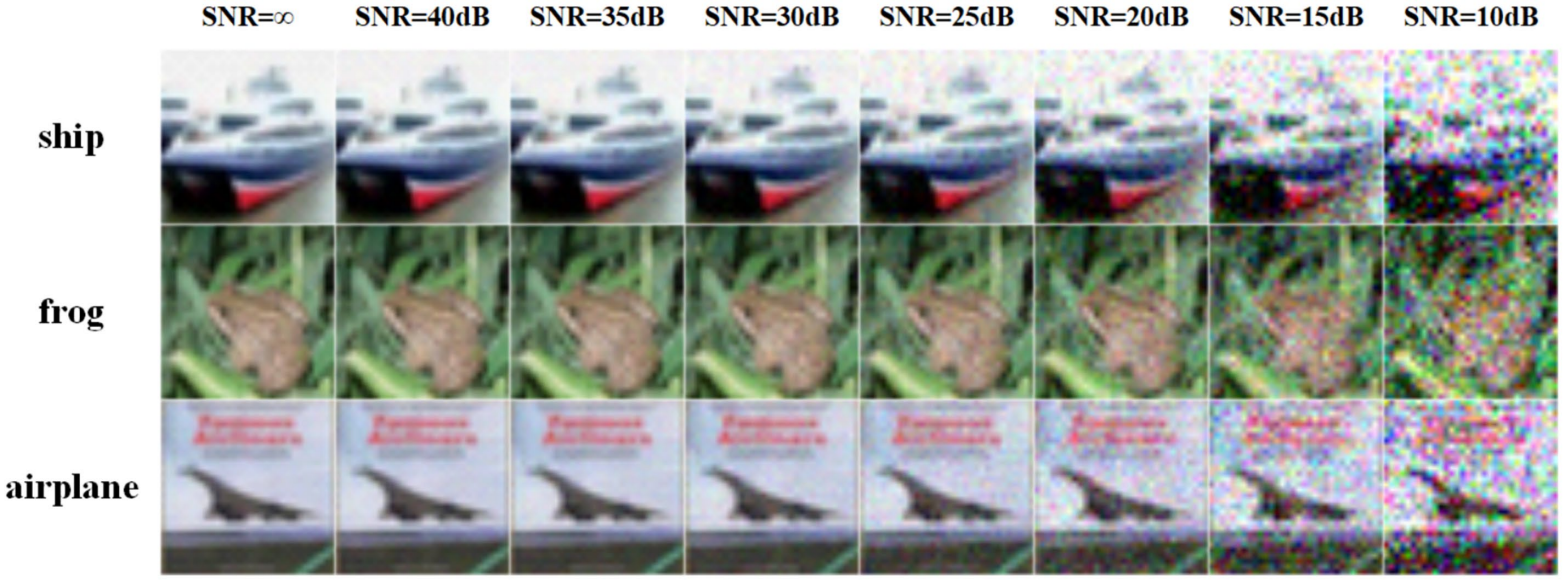
Impact of noise on image quality under different signal-to-noise ratio (SNR) conditions.

**Figure 8:**
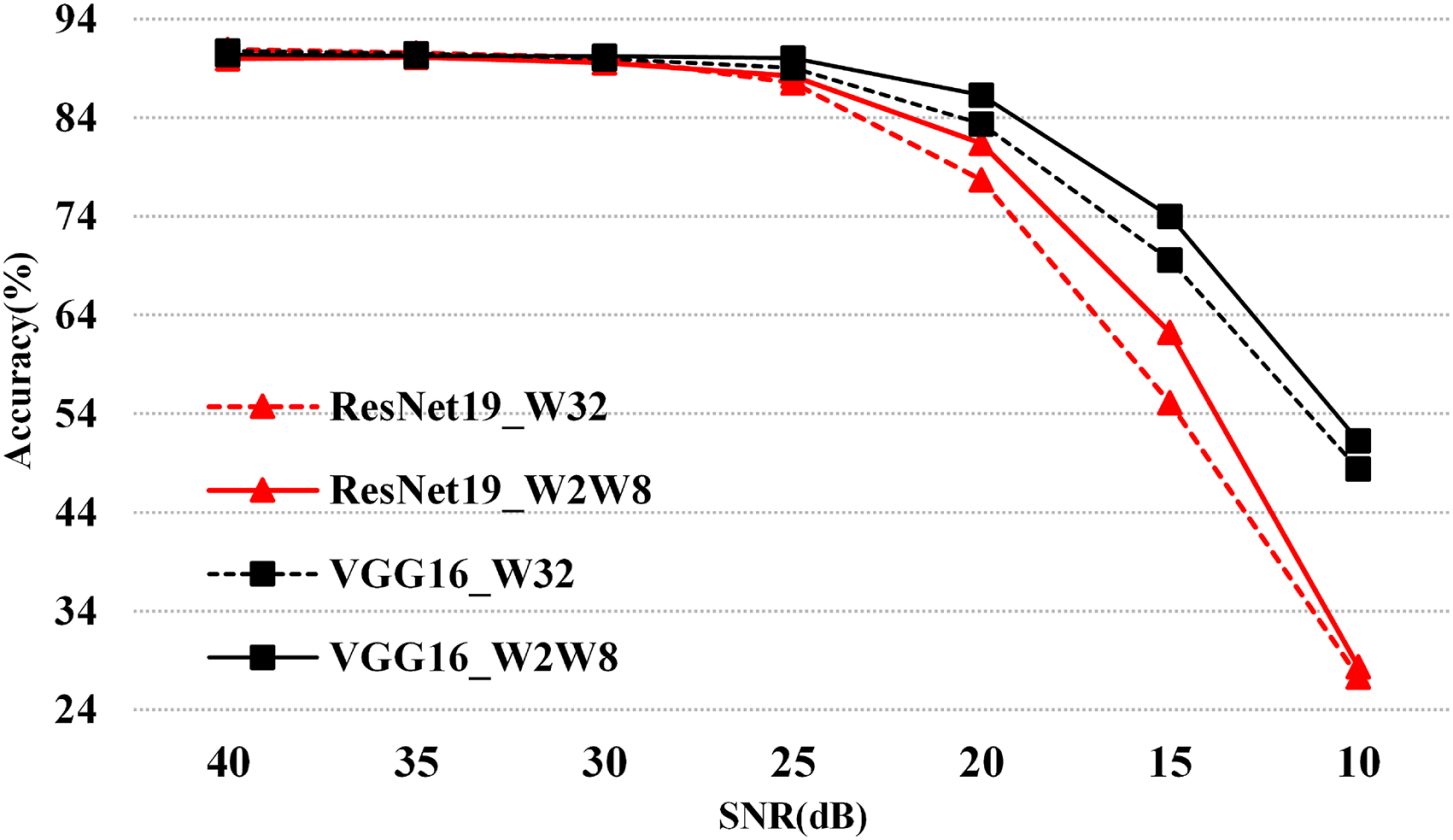
Robustness comparison of mixed-precision (w2w8) vs. full-precision (w32w32) on ResNet19 and VGG16 over CIFAR10 under varying SNR conditions.

To illustrate the effects of network architecture and quantization on accuracy under different SNR levels (as outlined in Figure 7), the corresponding results are presented in Figure 8. Specifically, at high SNR levels, the accuracy of the w2w8 model closely approaches that of the full-precision model. However, as the SNR decreases, the accuracy of the full-precision model shows a downward trend. For example, at an SNR of 15, the ResNet19 full-precision model achieves approximately 70% accuracy, whereas the w2w8 quantized model maintains a higher accuracy of about 74%. Similarly, for the VGG16 network at the same SNR, the full-precision model’s accuracy drops to around 55%, while the w2w8 model retains approximately 62%. These results show that, as the SNR diminishes, the proposed quantization method not only preserves the robustness of the model but exceeds that of the full-precision counterpart, showcasing improved robustness under noisy conditions.

### 4.5 Ablation studies

To investigate the compensatory effect of the proposed neurons on model accuracy, ablation experiments are conducted using the CIFAR-100 dataset with VGG11 and ResNet19 network architectures.

**Figure 9:**
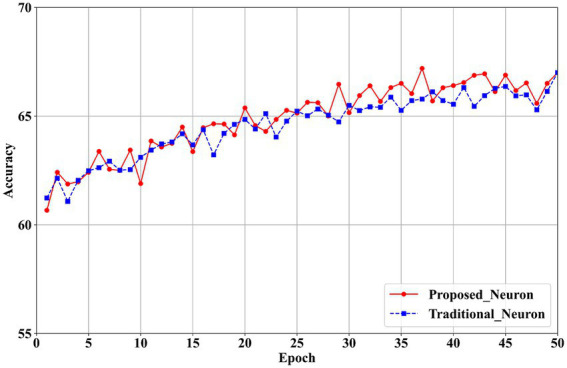
Training accuracy comparison between proposed and traditional neurons in VGG11 on CIFAR100.

**Figure 10:**
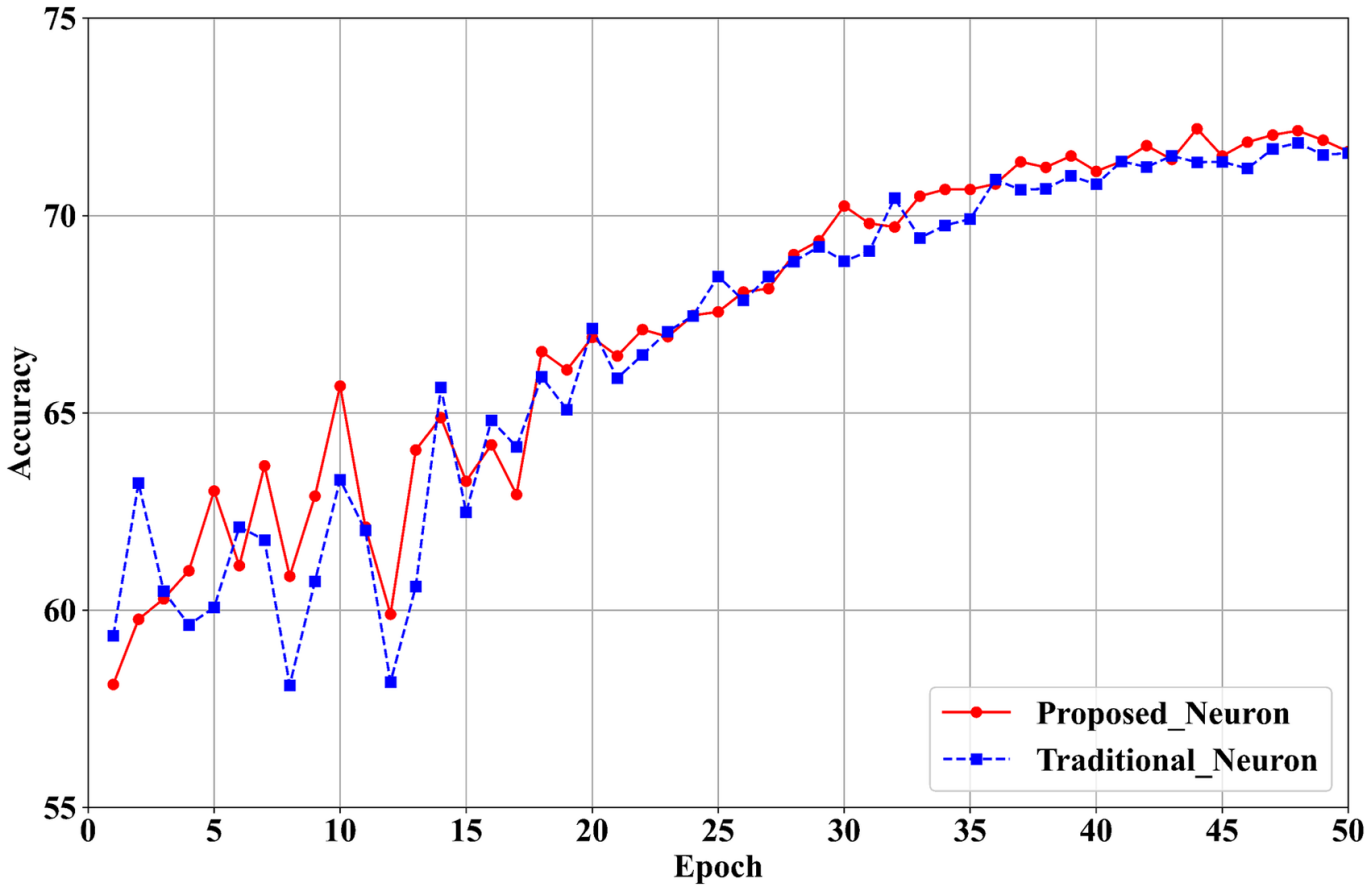
Training accuracy comparison between proposed and traditional neurons in ResNet19 on CIFAR100.

The experimental variables include the proposed neurons and traditional neurons (Guo et al., 2024). For the first layer, channel quantization is applied in both the experimental and control groups. In the subsequent layers, layer quantization (He and Cheng, 2018), (Wang et al., 2019) and traditional neurons (Guo et al., 2024) are used. The results are presented in Table 7, while accuracy trends over training epochs are illustrated in Figure 9 for VGG11 and Figure 10 for ResNet19.

**Table 7:** Ablation study comparing proposed versus traditional neurons in VGG11 and ResNet19 on CIFAR100 dataset.

Network	VGG11		ResNet19	
Traditional neuron	✓	×	✓	×
Proposed neuron	×	✓	×	✓
Accuracy	66.68	67.20	71.80	72.20

The results in Table 7 show that the proposed neurons enhance model performance. For the VGG11 model, classification accuracy increases from 66.68% with traditional neurons in the first layer to 67.20% with the proposed neurons, reflecting an improvement of 0.52%. In the ResNet19 model, accuracy in the first layer rises from 71.80% with traditional neurons to 72.20% with the proposed neurons, yielding a 0.40% improvement. These findings confirm that the proposed neurons effectively mitigate the performance loss associated with quantizing the first layer to 2 bits.

Figures 9, 10 illustrate that the proposed neurons outperform traditional neurons during training. Initially, the performance of the proposed neurons is approximately 1% lower than that of traditional neurons. However, their performance progressively exceeds that of traditional neurons. In the VGG11 model (Figure 9), the proposed neurons demonstrate greater stability than traditional neurons starting around the 25th epoch. Similarly, in the ResNet19 model (Figure 10), the proposed neurons consistently surpass traditional neurons beginning at approximately the 30th epoch.

Overall, the experimental results demonstrate the effectiveness of the neurons introduced in this paper. The proposed neuron successfully compensates for the performance loss associated with 2-bit quantization in the first layer, thereby enhancing the overall performance of the network. This improvement contributes to the development of a high-performance quantization model and offers valuable technical support for hardware unified computing architectures.

### 4.6 Hardware efficiency evaluation

In this section, we analyze the varying levels of parallelism in PE1. We use the decoupled PE architecture as the benchmark system, and its resource utilization will serve as the baseline for evaluating performance. This comparison will allow us to assess the effectiveness of different degrees of parallelism and their impact on resource utilization.

As shown in Table 8, the unified computing architecture exhibits low resource utilization. By integrating PE1 and PE2, critical logic resources are saved. Specifically, when the output parallelism of PE1 is low (1), the unified PE can save 1.22% of LUTs, 0.49% of flip flops (FFs), and 50% of digital signal processors (DSPs) compared to traditional decoupled PEs. When the output parallelism of PE1 is increased to 16, the unified PE achieves even greater resource savings, with reductions of 20.20% in LUTs, 10.59% in FFs, and 6.90% in DSPs.

**Table 8:** Resource comparison between decoupled PE and unified PE for first convolutional layer under different output parallelism.

*Pout* (1st layer)	Method	PE (*P_in_* × *P*_*ou*t_)	LUT	FF	DSP	FPS
1	Decoupled PE	PE1(3 × 1) & PE2(512 × 1)	149,642 (−0.00%)	142,212 **(**−0.00%**)**	12 **(**−0.00%**)**	320
	Unified PE	This work (512 × 1)	**147,818** **(−**1.22%**)**	**141,516** **(−**0.49%**)**	**6** **(−50%)**	
16	Decoupled PE	PE1(3 × 16) & PE2(512 × 1)	199,586 **(**−0.00%**)**	174,634 **(**−0.00%**)**	87 **(**−0.00%**)**	424
	Unified PE	This work (32 × 16 & 512 × 1)	**159,263** **(−20.20%)**	**156,137** **(−10.59%)**	**81** **(−6.90%)**	

Bold values indicate superior performance.

In addition, the resource-saving benefits of the unified computing architecture are amplified as the degree of parallelism increases. When the parallelism increased from 1 to 16, the savings jumped from 1.22 to 20.20% for LUTs and from 0.49 to 10.59% for FFs. This shows that, compared with the traditional architecture, the unified computing approach can effectively manage the growth of hardware resources in high-parallel real-time tasks.

It’s worth noting that the unified computing architecture aligns with the frames per second (FPS) of traditional architectures without compromising processing efficiency. This indicates that the unified computing architecture can achieve substantial resource savings while maintaining the same throughput levels.

In summary, the unified PE represents a more efficient hardware solution than the traditional discrete design. It not only reduces the logic resource utilization of FPGAs but also exhibits a significant scaling effect in high-parallel application scenarios.

In order to fully evaluate the effectiveness of the proposed SNN accelerator design, we conduct a detailed comparison with three advanced similar works on the CIFAR100 dataset. As shown in Table 9, our design exhibits excellent overall performance across several key metrics.

**Table 9:** Comparison between FPGA-based SNN accelerator and other SOTA designs.

Parameters	Chen et al. (2024)	Li et al. (2024)	Aung et al. (2023)	This work
Platform	Virtex-72000 T	Xczu3eg	VCU118	Virtex-7690 T
Neuron	LIF	LIF	LIF	LIF
Dataset	CIFAR100	CIFAR100	CIFAR100	CIFAR100
Clock Frequency	200	600	500	100
Model	VGG11	VGG11	VGG11	VGG11
Weight Bitwidth	8bit	8bit	8bit	2bit/8bit
Time Step	4	4	1	1
Accuracy	66.97%	64.3%	65.9%	67.38%
LUT	142,446	23 K	183 K	147,818
FF	124,619	–	–	141,516
Bram	355.0	103	289	326.5
DSP	1	256	2,881	6
Latency/Image (ms)	19	1.75	0.082	3.12
FPS	52	571	11.6 K	320
Power Consumption	1.562	6.2	29.8	0.982

Firstly, regarding classification accuracy, the proposed method achieves 67.38%, surpassing all comparison works. This outcome indicates that the T8HWQ scheme and the network architecture can be effectively deployed while maintaining high accuracy. Importantly, this level of performance is achieved at a single time step, without introducing any additional latency. Therefore, our method offers the dual benefits of low latency and high performance.

Secondly, the efficiency of this design is reflected in two key aspects: power consumption and resource utilization. On the Virtex-7690 T platform, the power consumption of the proposed accelerator is 0.982 W, representing a significant improvement over the 1.562 W reported in Chen et al. (2024). This advantage stems not only from the low-latency design achieved within a single time step but also from the implementation of first-layer ternary quantization technology. This technology reduces the first-layer multiplication operation to an equivalent addition operation, drastically decreasing dependence on DSPs. Specifically, our design requires only 6 DSPs, in contrast to 256 and 2,881 DSPs required by the schemes in Li et al. (2024) and Aung et al. (2023), respectively. These results demonstrate the applicability of our architecture in resource-constrained edge computing scenarios.

Regarding throughput, while the methods presented in Li et al. (2024) and Aung et al. (2023) achieve lower latency with higher clock frequencies (600 MHz and 500 MHz), these performance gains come at the cost of substantial power consumption and DSP resource utilization. In comparison, our design achieves an image processing delay of 3.12 ms and a throughput rate of 320 FPS at a clock frequency of only 100 MHz. The processing latency of our proposed method is approximately six times lower than that of Chen et al. (2024), despite the latter’s implementation being deployed for four time steps, which is four times that of our design. This indicates that our method possesses a highly competitive high-throughput characteristic.

In summary, this study demonstrates that through the software-hardware co-design strategy, the T8HWQ quantization method effectively facilitates the efficient reuse of computing resources. It achieves high accuracy at a single time step while maintaining low levels of power consumption and DSP resource utilization, making it well-suited for resource-constrained, low-latency edge computing scenarios.

### 4.7 Discussion on scalability for multi-timestep processing

Although the proposed design targets single-timestep scenarios for ultra-low latency processing, it can also scale to multi-timestep applications. Since our architecture does not rely on membrane potential states dependent on specific time steps, the simplest scaling approach involves adopting a temporal parallelism strategy (Yin et al., 2024), (Chen et al., 2024). In this approach, independent computing resources are allocated for each time step, thereby preserving extremely low latency.

This parallelism strategy highlights a fundamental trade-off in SNN accelerator design: the performance advantage of “temporal parallelism” versus the resource efficiency of “temporal serialism” (Narayanan et al., 2020). A more flexible and desirable approach involves a hybrid, configurable data flow (Lee et al., 2022) that dynamically balances latency and resource utilization based on specific application requirements. For example, for a task with six time steps (T = 6), the system can operate in a mode that processes time steps in parallel within a group and executes groups sequentially. This can be realized as three stages, each processing two time steps (T = 2) in parallel. Alternatively, it can run in two sequential stages, each processing three time steps (T = 3) in parallel.

The T8HWQ unified computing architecture proposed in this paper provides a robust foundation for achieving this goal. It minimizes hardware overhead while maintaining high performance through co-design of software and hardware, which makes it feasible to implement configurable data flows on resource-constrained platforms. In the future, we plan to develop and validate the implementation of this configurable data flow.

## 5 Conclusion

This paper addresses the critical issue of resource redundancy in SNN accelerators, a problem stemming from the inherent decoupling of quantization algorithms and FPGA computing units, by proposing a holistic software-hardware co-design methodology. Specifically, we propose a T8HWQ method and a channel-wise dual compensation strategy, which innovatively introduces channel-wise adaptive thresholds to compensate for quantization loss, and adopts a reparameterization method to reduce the quantization performance loss without increasing computation amount. In addition, this proposed method effectively reduces the hardware implementation overhead by supporting a unified computing architecture based on FPGA. Experimental results show that the quantization algorithm and the hardware design not only maintain the high performance of the network quantization, but also realize the resource reuse of computing units in one time step. This algorithm-hardware collaborative optimization scheme provides effective technical support for high-performance and low-latency processing in resource-constrained scenarios. On this basis, we will further explore the algorithm design and hardware architecture development of SNNs in more complex tasks such as object detection in the future (Chen et al., 2024).

## Data Availability

The original contributions presented in the study are included in the article/supplementary material, further inquiries can be directed to the corresponding author/s.
